# Reply: Internet chemotherapy information is of good quality: assessment with the DISCERN tool

**DOI:** 10.1038/bjc.2012.224

**Published:** 2012-06-07

**Authors:** E Davies, K-W Yeoh

**Affiliations:** 1 Department of Oncology/Haematology, Northampton General Hospital NHS Trust, Cliftonville, Northampton NN1 5BD, UK; 2 Department of Clinical Oncology, The Churchill Hospital, Oxford Radcliffe Hospitals NHS Trust, Old Road, Oxford OX3 7LJ, UK

**Sir,**

We thank Som and Gunawardana (2012) for their comments on our paper (Davies and Yeoh, 2012). Our study aimed to investigate the question of the impact of internet chemotherapy information (ICI) on patients and health professionals (HPs) and their perception of ICI. Som and Gunawardana have pointed out that the DISCERN tool is able to facilitate the assessment of the quality of websites. We note that DISCERN, like other instruments and methods, such as the HONcode and the JAMA benchmarks, have long been available for use by HPs (Silberg *et al*, 1997; Boyer *et al*, 1998; Charnock *et al*, 1999). Although these instruments are helpful in the systematic evaluation of ICI, they do not address the broader issues raised by our study, namely the need to (1) address inequalities of internet access; (2) maintain the quality of ICI (being an increasingly dynamic process); (3) bridge the gap of perception of ICI by patients and HPs; and (4) integrate ICI with traditional clinical consultation models.

Our study, Davies and Yeoh (2012), has shown that ICI is generally perceived by patients as a valuable information resource to augment information that is traditionally obtained through HPs. Although the HPs in this study had some concerns regarding the possible detrimental effect to some patients and their ability to interpret internet information, as reiterated by Som and Gunawardana, the majority recognised and supported the need for patients to retrieve ICI to improve their understanding of chemotherapy treatment. We highlighted discrepancies that exist between HPs’ perception and the patients’ needs with regard to the ICI-seeking behaviour. The reasons for the discrepancies are multifactorial and emphasise a need for HPs to work more closely with the patients in addressing their concerns. The UK’s National Cancer Action Team’s ‘Patient Information Prescription’ Cancer patient information pathways (2012) is a recently developed web-based tool, which is now gaining popularity as a patient education resource. This offers a comprehensive approach to the disease and treatment information, enabling HPs to provide standardised peer-reviewed information to patients. It also allows them to direct patients to high-quality websites according to individual needs. Perhaps the best application of ICI should be its integration to work synergistically with current traditional consultation models to enhance patient experience and provide additional information in conjunction with that provided by HPs.

Given the potential impact of the ICI and the dynamic nature of web-based resources, it will be important to address the different ways in which HPs and patients perceive ICI. It is also important to focus on the maintenance of the quality of ICI rather than just assessing its quality. Several other issues such as inequalities of internet access will also need to be considered if ICI is to be integrated meaningfully with traditional consultation models.
